# Quantitative Analysis of Barriers to Clubfoot Treatment Experienced at a Tertiary Care Institute in India

**DOI:** 10.7759/cureus.25782

**Published:** 2022-06-09

**Authors:** Saral J Patel, Manish V Shah, Mitul Mistry, Ravindra Zala, Himanshu Panchal, Mukund Prabhakar, Bhautik Modi

**Affiliations:** 1 Department of Orthopaedics, Dr. N.D. Desai Faculty of Medical Science and Research, Nadiad, IND; 2 Department of Orthopaedics, Gujarat Medical Education & Research Society (GMERS) Medical College, Ahmedabad, IND; 3 Department of Orthopaedics, Gujarat Medical Education & Research Society (GMERS) Medical College, Gandhinagar, IND; 4 Department of Orthopaedics, B.J. Medical College, Ahmedabad, IND; 5 Department of Community and Family Medicine, All India Institute of Medical Sciences, Rajkot, IND

**Keywords:** india, quantitative analysis, dropout rate, compliance, barriers, clubfoot

## Abstract

Introduction: The Ponseti method of treating clubfeet is considered the gold standard. However, there are specific barriers to implementing the Ponseti method for clubfoot treatment, especially in developing countries like India.

Methods: This is a retrospective study on patients who underwent the Ponseti method for clubfoot treatment at a tertiary care hospital in India. A total of 110 patients were enrolled for this study and were interviewed at the initiation of treatment and at each follow-up to identify the barriers, and their correlation to dropout rate was analyzed.

Results: On applying binary logistic regression, which shows the cumulative effect of all variables, the effect of the parent accompanying the patient was significant on compliance and dropout rate.

Conclusion: Informed parents play a significant role in compliance with the treatment. The study results can help formulate an action plan to improve adherence to the Ponseti method for treating clubfoot throughout India and other developing countries.

## Introduction

Clubfoot is a complex congenital deformity with a component of forefoot adduction, midfoot cavus, hindfoot varus, and ankle equines [1]. It can be idiopathic or a part of a syndrome like arthrogryposis multiplex congenita (a congenital condition with stiffness in multiple joints). As per the 2016 National Institution for Transforming India (NITI) Aayog Data, India has an annual birth rate of 20.4 births per 1000 people [2]. Considering the population of India, the annual birth rate, and the incidence of clubfeet as 1/1000 live births, the estimated number of infants born with clubfoot is approximately 27,000 per year. The deformity, if left untreated, can result in permanent physical disability, which in turn can result in a social, economic, and psychological burden on the individual and family.

Currently, the treatment of clubfeet by the Ponseti method is considered the gold standard [3]. The main components of the treatment are serial manipulation and casting followed by a percutaneous tendoachilles tenotomy and a foot abduction brace. The treatment can achieve complete correction in >95% of patients [4-11]. The treatment is relatively cost-effective compared to surgery, making it an ideal choice for developing countries like India.

Various studies worldwide have highlighted specific barriers to implementing the Ponseti method for clubfoot treatment. These barriers include poverty, cost of treatment, cost of traveling, caregiver's compliance to the treatment, and the parent responsible for the treatment, to list a few [10,12-17]. The purpose of this study is to analyze the effect of these barriers on the dropout rates observed with the Ponseti method of clubfoot treatment.

## Materials and methods

A retrospective study was conducted at a tertiary care government-funded hospital in the western part of India, after obtaining approval from the institutional ethics committee. The patients who underwent the Ponseti method for clubfoot at the hospital were identified. All patients who presented with idiopathic clubfoot at any age and completed the entire treatment (completed three-four years of night bracing) or dropped out of the treatment were included in the study. These patients were followed up and proper treatment was administered at the institute.

**Figure 1:**
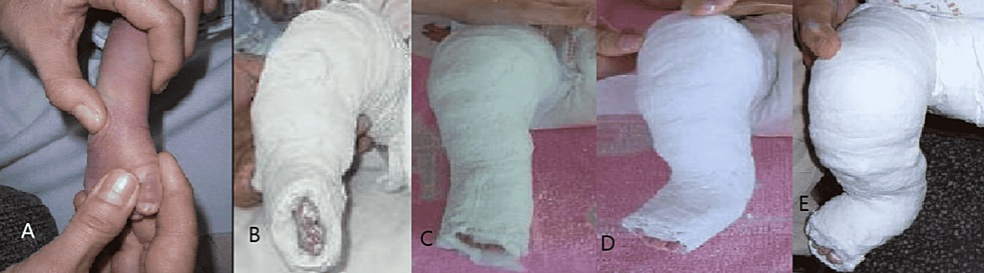
The Ponseti method of clubfoot correction. A: Technique of manipulation and correction of the deformity prior to applying a cast. B-E: Serial correction of the clubfoot deformity using the Ponseti method of casting.

At the hospital, there is a dedicated clubfoot clinic that operates thrice a week to reduce the waiting period at the hospital. All patients with clubfoot are treated by trained physicians using the Ponseti method, with strict adherence to the protocol as shown in Figure 1.

A pretested semi-structured questionnaire was filled for each patient by a physician or post-graduate trainee fluent in English and regional language by personal interview during the initial visits after obtaining informed written consent of their parents. This questionnaire includes sociodemographic variables like age, education status of parents (the highest educational degree amongst the parents), socio-economic status, earning parent, parent accompanying the patient (the parent who accompanied the patient at the majority of the visits), cultural beliefs of the parents (belief of parents that the clubfoot deformity is caused by the will of God, punishment from God, birth at the time of an eclipse, etc.), rural/urban area, distance to the clinic, and daily wage lost during the visit (average of the daily wage lost during the entire treatment was considered for analysis). As the state funds the entire treatment, the cost of the treatment is not recorded for these patients.

Data of these patients were extracted from the clubfoot clinic. After the end of treatment of all patients, different variables in patients who completed the treatment and patients who did not complete the treatment were evaluated and barriers were identified. Thus, variables associated with dropouts were identified and compared.

Patients who completed the treatment were considered compliant. Patients were considered dropouts from treatment if they missed three consecutive planned follow-up visits during the casting or bracing phase [16]. All patients with clubfoot as a part of a syndrome or secondary to other cause, recurrence after surgery for clubfoot, or patients under treatment were excluded from the study. For the calculation of the sample size, a pilot study was conducted and a variable (distance from the hospital) was evaluated. As per the pilot study, 7.8% of patients traveled a distance of more than 50 km for the treatment among dropout patients. Using this variable, the calculated sample size using these data by OpenEpi software was 110 at a 95% confidence interval.

Continuous variables were summarized by using mean and standard deviation, and categorical values were summarized by using frequencies and percentages. Statistical analysis was performed using Microsoft Excel 2019 (Redmond, WA) and R software (R Foundation for Statistical Computing, Vienna, Austria). Chi-square test and binary logistic regression analysis were used to detect the correlation between the barriers and dropout rate, and the result was considered significant if p < 0.05.

## Results

Most of the patients were between one and two years of age at the initiation of treatment. The mean age of the patients was 1.8 years. The mean number of casts applied before tenotomy was 7.29 ± 2.51. The demographic and socioeconomic parameters for the 110 patients are outlined in Table 1.

**Table 1:** Analysis of the demographic and socioeconomic parameters (N = 110)

Parameters		N (%)
Age of child at the start of treatment	<1 year	15 (13.64)
	1-2 years	55 (50)
	2-5 years	35 (31.82)
	>5 years	5 (4.55)
Parent accompanying the child	Both	87 (79.09)
	Father	10 (9.09)
	Mother	13 (11.82)
Education status of the parent	Graduate	8 (7.27)
	Higher secondary	61 (55.45)
	Secondary	34 (30.91)
	Primary	1 (0.91)
	Illiterate	6 (5.45)
Distance to clinic	<10 km	55 (50)
	10-15 km	7 (6.36)
	15-20 km	1 (0.91)
	>20 km	47 (42.73)
Rural/urban area coverage	Rural	43 (39.09)
	Urban	67 (60.91)

**Figure 2:**
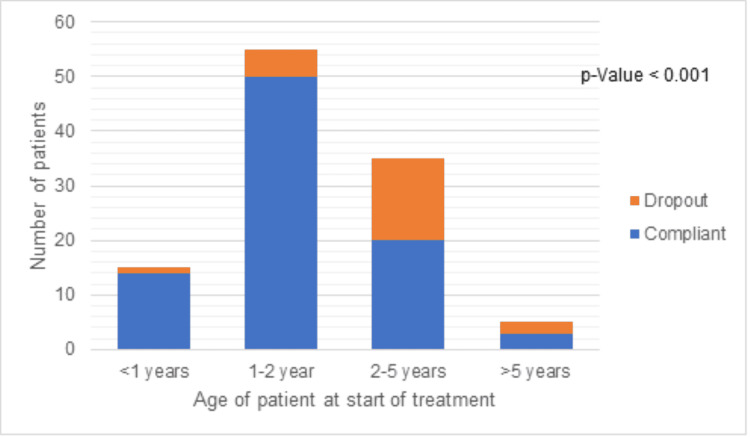
Compliance according to the age of the patient at the start of the treatment

**Figure 3:**
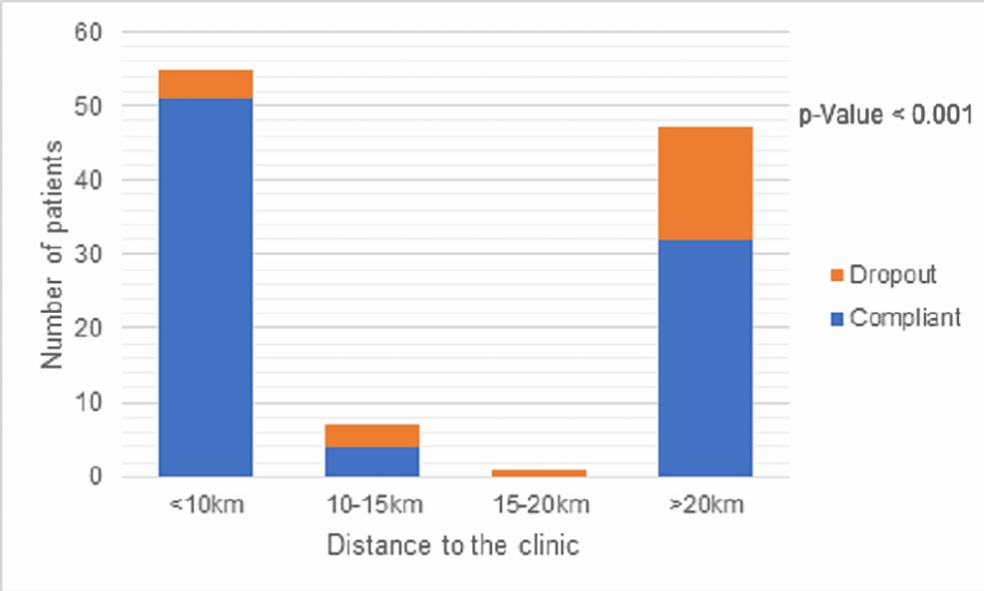
Compliance according to the distance to the clinic

The relation between dropout rates and different parameters was measured using the chi-square test, and the result is outlined in Table 2 and graphically represented in Figures 2, 3. Barriers that had a significant relation (p < 0.05) with the dropout rate were age, education status of parents, socioeconomic status, parent accompanying the patient, cultural belief, rural/urban area, distance to the clinic, understanding of clinician advice, and motivation to complete the treatment.

**Table 2:** Compliance according to different variables * Statistically significant observation with p < 0.05 as per the chi-square test.

	Compliant (completed the treatment)	Dropout	Chi stats	P-value
Age of patient at the start of treatment				
<1 year	14	1	17.78	0.00*
1-2 years	50	5		
2-5 years	20	15		
>5 years	3	2		
Parents having knowledge of clubfoot and its treatment on the first visit				
Yes	59	16	0.03	0.87
No	28	7		
Having cultural belief				
Yes	0	7	22.74	0.00*
No	87	16		
Daily wages lost due to clinic attendance				
>1000	1	1	3.39	0.18
100-500	35	13		
500-1000	51	9		
Parent accompanying the child				
Both	70	17	6.58	0.04*
Father	5	5		
Mother	12	1		
Education status of parent				
Graduate	7	1	0.42	0.02*
Higher secondary	49	12		
Secondary	29	5		
Primary	0	1		
Illiterate	2	4		
Socioeconomic status				
High	3	0	10.08	0.01*
Medium	80	17		
Low	4	6		
Earning parent				
Both	0	1	10.03	0.08
Father	87	22		
Rural/urban area coverage				
Rural	28	15	8.34	0.00*
Urban	59	8		
Distance to clinic				
<10 km	51	4	14.66	0.00*
10-15 km	4	3		
15-20 km	0	1		
>20 km	32	15		
Understanding clinician advice fully				
Yes	87	15	26.31	0.00*
No	0	8		
Motivation for completion of treatment				
Yes	87	16	22.74	0.00*
No	0	7		

Binary logistic regression was applied to the dataset, and cumulative effects of all variables were assessed as outlined in Table 3. On using enter model, the R square value was 0.832. This shows that around 83% of the variance was explained by this model. On applying the Hosmer-Lemeshow test, the significance level was 1.00, indicating that the model can be used for interpretation. As per Table 3, patients are more likely to be compliant if both the parents attend with the patient during each visit.

**Table 3:** Result of binary regression analysis

Enter model	-2 log-likelihood	Nagelkerke R square					
	28.953	0.832					
Hosmer-Lemeshow test	Chi-square	df				Sig.	
	0.443	8				1.000	
Variable	B	S.E.	Wald	df	Sig.		Exp(B)
Attendant with patient			6.046	2	0.049		
Both parents	5.744	2.336	6.046	1	0.014		312.194
Only father	-1.77	9503.444	0.000	1	0.999		1.977

Thus, on applying the chi-square test, the effect of most of the variables was significant on the compliance of the patient. However, applying binary logistic regression, which shows the cumulative effect of all variables, the effect of the person accompanying the patient is significant on compliance.

## Discussion

Various studies in low and middle-income countries such as Malawi, Uganda, China, Bangladesh, and India have used questionnaires to identify barriers to the successful implementation of clubfoot treatment programs [10,12-17]. Despite the differences in population, cultures, and resources, the barriers identified are similar in all these countries.

In Uganda, Malawi, China, Bangladesh, and India, poverty has been observed as a barrier to clubfoot treatment [10,12-17]. In our study, patients from lower and middle socioeconomic conditions had a higher dropout rate. The cost of treatment was identified as a barrier to a clubfoot treatment program in China [10]. The clubfoot program at the hospital in this study is entirely funded by the government, similar to Uganda [15]. So, the patient does not have to spend any money on casts or braces. Indirect financial burden in the form of daily wages lost on the day of attending clinics was considered as a potential barrier to the treatment. However, there was no relation between the financial burden in wages lost and the dropout rate.

Parents who were illiterate or had received only primary education had a higher dropout rate. Also, parents with strong cultural beliefs regarding clubfoot had a higher dropout rate. Parents' knowledge about clubfoot and its treatment before the start of treatment did not correlate with the dropout rates, similar to the studies in Uganda and Malawi [12,14,15]. However, parents who understood the clinician’s advice and were motivated to complete treatment had a significantly lower dropout rate. Education and strong beliefs may hinder parents from completely understanding the clinician’s advice and affect compliance with treatment. In the present study, the patient's age was identified as a barrier to clubfoot treatment, with higher dropout rates observed in older children. Prolonged treatment duration for older children may have affected their compliance to the treatment and resulted in an increased dropout rate.

Patients from rural localities and those who traveled more than 50 kilometers to reach the clinic also had a higher dropout rate. All these factors make it difficult for parents to take a day off from work and incur the cost of traveling such a considerable distance to attend the clinic at regular intervals. A similar finding was observed in Uganda and China as well [10,15].

In the present study, most of the children were accompanied by both the parents to the hospital and had a good compliance rate. It was observed that the child brought to the hospital by their father solely had a high chance of dropping out of the treatment. In Uganda and Malawi, it was observed that the mothers are primarily responsible for treating the child with clubfoot [12,14,15]. In China, most children are accompanied by both parents, similar to our study [10]. On considering the cumulative effect of all variables, the effect of the person accompanying the patient is significant on the compliance to treatment, indicating a vital role of both parents in completing the treatment. In India, we have observed that the father is usually the earning member of the family while the mother is the caretaker of the child. We believe it becomes difficult for the father to take care of the child and simultaneously earn for the family without the support of the mother, resulting in a higher dropout rate.

Based on this study, it is recommended to establish government-funded clubfoot clinics at the district and taluka level, which will help to serve the rural and urban locality, reduce the traveling distance, and hence the time spent on getting the treatment. Such clinics should be organized at least twice or thrice a week, equipped with physicians trained in the Ponseti method, nurses, and trained counselors who can educate the parents on the importance of each step of treatment and follow up with them. Another advantage of having a clubfoot clinic is that it works as group therapy for parents. Parents of new patients can see the correction achieved in other children and get motivated to complete the treatment.

Training the ancillary medical staff who work in the rural areas can help early identification of the deformity, timely referral to the nearest clubfoot clinic, and counsel the parents to complete the treatment and ensure regular follow-up. Educating the parents through pictures and videos might help when parents are illiterate or have cultural beliefs. A collaboration between non-government organizations and government agencies can help achieve these goals.

However, this study has limitations as it is based on a small population group in Western India. The barriers were identified based on the population who attended the clinic, and it is not known if they are the same as those experienced by the population who have not availed treatment. A multi-centric study can help better identify the barriers.

## Conclusions

The study identified the barriers to clubfoot treatment, which significantly impacted compliance and dropout rates from the treatment. The barriers identified are a low level of education among the parents, lower socioeconomic status, rural locality, distance to the clinic, cultural beliefs, failure to understand clinician's advice, lack of motivation to complete treatment, child's older age, and parent accompanying the child.

However, considering the cumulative effect of all variables, the effect of the parent accompanying the patient is the single most important factor associated with compliance with treatment. The study results can help formulate an action plan to improve adherence to the Ponseti method for treating clubfoot throughout India.
